# A cross-site antimicrobial resistance surveillance system using semantic web technologies

**DOI:** 10.1186/1753-6561-5-S6-O35

**Published:** 2011-06-29

**Authors:** D Teodoro, E Pasche, D Vishnyakova, B De Vloed, K Depraetere, P Ruch, C Lovis

**Affiliations:** 1 SIMED, University and University Hospitals of Geneva, Geneva, Switzerland; 2 AGFA Healthcare, Ghent, Belgium; 3 University of Applied Sciences, Geneva, Switzerland

## Introduction / objectives

Bacterial resistance to drugs has reached alarming levels but useful cross-site monitoring systems to track resistance evolution are lacking. In this paper we present the *TrendMon* surveillance system, a platform for querying, integrating and visualising antimicrobial resistance information.

## Methods

TrendMon is developed within the EU FP7 DebugIT (Detecting and Eliminating Bacteria Using Information Technology) project. It builds on another DebugIT component, the virtual Clinical Data Repository (vCDR), which integrates clinical information systems, using RDF (Resource Description Format) and SPARQL (SPARQL Protocol and RDF Query Language) to formally describe and access sources respectively. It also exploits biomedical domain ontologies, such as NEWT and WHO-ATC, to formalise, normalise and enrich the data content.

## Results

Datasets covering microbiology test and antibiotherapy information from 2000 to 2009, from seven healthcare institutes were shared within the consortium. A set of clinical questions of public health interest was proposed to assess the system's ability to track resistance trends from heterogeneous sources. In this limited scope, TrendMon managed to automatically integrate and extract trends from six out of seven hospitals. Furthermore, it allowed generating views by drug (anatomical, therapeutic and chemical axis) and bacteria (genus, taxon) clusters.

## Conclusion

TrendMon is a powerful tool for monitoring bacterial resistance patterns. The main challenge found in the design was to represent formally the data sources. The next step is to integrate the proof of concept in real time clinical information systems. Ultimately, the clinical meaning of the extracted trends needs to be validated.

## Disclosure of interest

None declared.

